# Bisphenol A and Bisphenol S Induce Distinct Transcriptional Profiles in Differentiating Human Primary Preadipocytes

**DOI:** 10.1371/journal.pone.0163318

**Published:** 2016-09-29

**Authors:** Jonathan G. Boucher, Rémi Gagné, Andrea Rowan-Carroll, Adèle Boudreau, Carole L. Yauk, Ella Atlas

**Affiliations:** 1 *In Vitro* Molecular Toxicology Laboratory, Environmental Health Science and Research Bureau, Health Canada, 50 Columbine Driveway, Ottawa, Canada; 2 Mechanistic Studies Division, Environmental Health Science and Research Bureau, Health Canada, 50 Columbine Driveway, Ottawa, Canada; INRA, FRANCE

## Abstract

Bisphenol S (BPS) is increasingly used as a replacement plasticizer for bisphenol A (BPA) but its effects on human health have not been thoroughly examined. Recent evidence indicates that both BPA and BPS induce adipogenesis, although the mechanisms leading to this effect are unclear. In an effort to identify common and distinct mechanisms of action in inducing adipogenesis, transcriptional profiles of differentiating human preadipocytes exposed to BPA or BPS were compared. Human subcutaneous primary preadipocytes were differentiated in the presence of either 25 μM BPA or BPS for 2 and 4 days. Poly-A RNA-sequencing was used to identify differentially expressed genes (DEGs). Functional analysis of DEGs was undertaken in Ingenuity Pathway Analysis. BPA-treatment resulted in 472 and 176 DEGs on days 2 and 4, respectively, affecting pathways such as liver X receptor (LXR)/retinoid X receptor (RXR) activation, hepatic fibrosis and cholestasis. BPS-treatment resulted in 195 and 51 DEGs on days 2 and 4, respectively, revealing enrichment of genes associated with adipogenesis and lipid metabolism including the adipogenesis pathway and cholesterol biosynthesis. Interestingly, the transcription repressor N-CoR was identified as a negative upstream regulator in both BPA- and BPS-treated cells. This study presents the first comparison of BPA- and BPS-induced transcriptional profiles in human differentiating preadipocytes. While we previously showed that BPA and BPS both induce adipogenesis, the results from this study show that BPS affects adipose specific transcriptional changes earlier than BPA, and alters the expression of genes specifically related to adipogenesis and lipid metabolism. The findings provide insight into potential BPS and BPA-mediated mechanisms of action in inducing adipogenesis in human primary preadipocytes.

## Introduction

Bisphenol A (BPA) is an industrial chemical used in the manufacture of polycarbonate plastic found in a number of consumer products such as thermal paper, canned foods and epoxy resins [1]. Human exposure to BPA is ubiquitous, and measurable amounts of BPA were present in the urine or blood in the general population [2, 3]. Due to recent regulatory restrictions and public pressure, both in Canada and in other countries, bisphenol S (BPS) is now commonly used as a substitute for BPA in the manufacture of polycarbonate plastic and is found in similar consumer products [4–6]. Like BPA, BPS has also been detected in the environment and indoor dust samples [7, 8] and human exposure to BPS has been confirmed through urine analysis [8, 9].

Many studies to date have linked BPA exposure to human negative health outcomes including breast cancer, reproductive disorders, heart disease and obesity [10–13]. In contrast, the effects of BPS on endocrine function and human health have not been as extensively studied. Emerging evidence suggests that BPS may have endocrine disrupting effects, much like BPA [14–16]. We and others have previously shown that BPA [17–20] and its metabolite BPA-glucuronide (BPA-G) induce lipid accumulation and adipogenesis *in vitro* [19]. Moreover, we have also reported that BPS is adipogenic in human primary preadipocytes, where it induced lipid accumulation and expression of adipogenic genes both at high and at environmentally relevant concentration [21]. In addition, we and others have shown that BPS treatment increased lipid metabolism, glucose uptake and adipogenesis in the murine 3T3L1 preadipocyte cell line [22, 23]. Further, we have also reported that BPS was more potent than BPA in inducing adipocyte-related genes, and that BPS but not BPA was a partial PPARγ agonist [23]. Despite the cumulative data showing BPS and BPA-mediated effects the mechanisms of action by which these chemicals affect endocrine function, lipid accumulation and adipogenesis, in particular, remain to be determined.

The objective of the current study was to compare the gene expression profiles of differentiating human primary preadipocytes in response to BPA and BPS to identify potential mechanisms of actions involved in BPA- and BPS-induced adipogenesis. To do this, human primary preadipocytes were exposed to 25μM BPA, 25μM BPS or DEX for two or four days, alongside concurrent solvent controls. Poly-A RNA sequencing was used to derive gene expression profiles. The results provide important molecular insight into the mechanisms leading to altered adipogenesis. This study presents the first comparison of the gene expression profiles of BPA- and BPS-treated preadipocytes early in adipogenesis and provides insight into mechanisms of action of both chemicals in the adipogenic process providing information on potential metabolic effects and potential obesity related health risk.

## Materials and Methods

### Adipocyte differentiation

Human subcutaneous primary preadipocytes (Zenbio, Inc., Research Triangle Park, NC, USA) from five different female donors with body mass indexes < 25 and who gave informed consent were differentiated in Preadipocyte Medium (ZenBio, Inc., Research Triangle Park, NC, USA) containing 33 μM biotin, 17 μM pantothenate, 100 nM insulin (Roche Diagnostics, Laval, QC, Canada), and 500 μM of 3-isobutyl-1-methylxanthine (IBMX) from day 0 to day 4 and 5 μM troglitazone from day 2 to day 4 (all from Sigma-Aldrich, Oakville, ON, Canada). In addition, cells were treated with vehicle control (ethanol), 25 μM BPA, 25 μM BPS, previously found to be optimal concentrations for the induction of adipocyte differentiation [18, 21, 23] or 1 μM dexamethasone (DEX), as a positive control (Sigma-Aldrich, Oakville, ON, Canada) every two days for 2 or 4 days as indicated. Ethics approval for the use of human cells was obtained by the Health Canada Research Ethics Board.

### RNA isolation

Total RNA were extracted from differentiating cells treated as described above using the RNeasy Kit and genomic DNA was eliminated using the RNase-Free DNase Kit (both from Qiagen, Toronto, ON, Canada). RNA was quantified using a NanoDrop 2000 (Thermo Scientific, Waltham, USA) and RNA quality was determined using a BioAnalyzer (Agilent Technologies, Santa Clara, USA). RNA samples with A260/A280 ratios > 1.8 and RNA integrity numbers (RIN) > 8.0 were used.

### Ion Proton™ Sequencing (RNA-Seq)

Total RNA (1μg) from four treatment groups of preadipocytes from five unique donors were used for RNA-Seq. Poly-A RNA enrichment (DynaBeads® mRNA DIRECT Micro Kit) was performed for each sample on 1 μg of total RNA. The Ion Total RNA-Seq Kit v2 was used to fragment and prepare the sample libraries from poly-A enriched samples. The 3’-end barcode adapters provided in the Ion Xpress™ RNA-Seq Barcode Kit were ligated to the ends of the fragmented libraries (each PCR product receiving its own unique barcode). Libraries were then amplified using the Platinum® PCR SuperMix High Fidelity. Each amplified library was quantified/qualified using the High Sensitivity D1000 ScreenTape Kit and the Agilent® 2200 TapeStation Instrument. Aliquots of each library were pooled together for a total final concentration of 50 pM. Emulsion PCR (em-PCR) and chip loading was performed on the Ion Chef with the Ion P1 HI-Q Chef kit. The chips (P1 v3) were run on the Ion Proton using the HI-Q chemistry. The Proton™ Torrent Server version 4.3 interpreted the sequencing data and generated unaligned BAM files for each barcoded sample. Reads were trimmed to remove low quality read prefixes, then aligned to the reference genome (GRCh38v77) using Star [24] and Bowtie [25]. Following alignment, gene counting was performed with HT-Seq count (http://www-huber.embl.de/users/anders/HTSeq/doc/count.html) with the m parameter set to “intersection-nonempty” using the Ensembl GTF annotation (GRCh38v77). The table of counts was then imported into R where genes with a total count less than 0.5 read per million reads per dose group were removed from further analyses. The EdgeR [26] package was used for the analysis by normalizing with TMM [27] and calculating differentially expressed genes using the GLM function. The donor ID was blocked in the design matrix to reflect the experimental design. The data is publically available from Sequence Read Archive (www.ncbi.nlm.nih.gov/Traces/sra/sra.cgi?view=studies; BioProject ID: SRP072037).

### Biological and pathway analyses

All genes with a false discovery rate (FDR) p < 0.05 and fold-change > +/- 1.5-fold up- or down-regulated compared to matched controls were considered significantly differentially expressed and selected for further analyses. Biological functions, pathways and upstream regulatory molecules/networks were analyzed using Ingenuity Pathway Analysis (IPA) (Ingenuity Systems, Redwood City, USA) as further described. The differentially expressed genes were uploaded into Ingenuity Pathway Analysis (IPA) (Ingenuity Systems, Redwood City, USA) for functional analysis. Within IPA, a standard analytical workflow was used to identify the disease and biological functions (containing high quality gene ontology information and curated lists of genes from the literature linked to specific biological functions and diseases), and IPA canonical pathways (curated pathways with specific directionality shown) that were over-represented (enriched) within the dataset and upstream regulator networks were analyzed using IPA (Ingenuity Systems, Redwood City, USA). The significance of the association between the gene expression dataset and the pathways/ and functions in IPA was measured using Fischer’s exact test (deemed significant if p ≤0.05). Regulatory networks (generated de novo in IPA using the input data and curated connectivity across molecules, genes, pathways, and other networks (etc.) in IPA’s ‘Global Molecular Network’) were visualized to explore connectivity across the molecules in the data set. IPA networks are ranked and assigned a p-score based on a Fisher’s exact test using the number of genes in the network and the number of focus genes in the network relative to IPA’s ‘Global Molecular Network’ (ref: https://www.ingenuity.com/wp-content/themes/ingenuity-qiagen/pdf/ipa/IPA-netgen-algorithm-whitepaper.pdf). IPA was also used to predict upstream modulators (including transcription factors, cytokines and chemical exposures) that are consistent with the altered patterns of transcription observed and that might be driving the expression changes. These analyses provide insight into different aspects of the toxicological response for the chemical treatments, enabling an evaluation of significant biological perturbations, what may be the primary factors and signalling molecules regulating the observed transcriptional changes. These reflect IPA derived networks used to demonstrate the interaction of molecules in the gene lists and assess what may be major regulators of effects and biological outcomes. The significance of the interactions between different nodes is not shown in the network. Instead, the derived networks are ranked and assigned a p-score (defined as–log(p-value)) based on a Fisher’s exact test using the number of genes in the network and the number of focus genes in the network relative to IPA’s ‘Global Molecular Network’ (ref: https://www.ingenuity.com/wp-content/themes/ingenuity-qiagen/pdf/ipa/IPA-netgen-algorithm-whitepaper.pdf).

### Quantitative RT-PCR

RNA (1 μg) was reverse transcribed into cDNA using the iScript Advanced cDNA Synthesis Kit (BioRad). Sample cDNA was amplified in a CFX96-PCR Detection System using the iQSYBR SsoFast EvaGreen Supermix (BioRad). The primer pairs for each gene target were: phosphoenolpyruvate carboxykinase 1 (*PCK1)*: Forward-ATCCACATCTGTGACGGCTC, Reverse-GGGTCAGTGAGAGCCAACC; cell death-inducing DFFA-like effector C (*CIDEC)*: Forward-AACGCAGTCCAGCTGACAAG Reverse-CACTGACACATGCCTGGAGA; fatty acid binding protein 5 (*FABP5)*: Forward-CATGAAGGAGCTAGGAGTGGG, Reverse-GCTCTCAGTTTTTATGGTGAGGT; glycerol-3 phosphate dehydrogenase 1 (*GPD1)*: Forward-ACATTGGAGGCAAAAAGCTGAC, Reverse-TGGGACAGCCACCACATTT; perilipin 4 (*PLIN4)*: Forward-CATTTGAACACGCGGTGAGC, Reverse-GCTGGGCCTTTTCAATCAGC; adiponectin (*ADIPOQ)*: Forward-ACCAGAGGAGGAGTAGGTTTGT, Reverse-TCATCAGTTTGCTTGTCTCTTCT; and β-Actin (*ACTB)*: Forward-GACTTCGAGCAAGAGATGGC, Reverse-CCAGACAGCACTGTGTTGGC. Primer efficiencies were ≥90% and specificity was confirmed by sequence blast and melting curve analysis. All target gene transcripts were normalized to *ACTB* and the comparative *C*_T_ (ΔΔ*C*_T_) method was used for data analysis. Statistical significance was analysed by one-way ANOVA with Holm-Sidak post-test analysis using SigmaPlot 12.5 (SigmaPlot, San-Jose, CA, USA).

## Results

### Expression profiles of BPA- BPS- and DEX-treated preadipocytes

**Fig 1:**
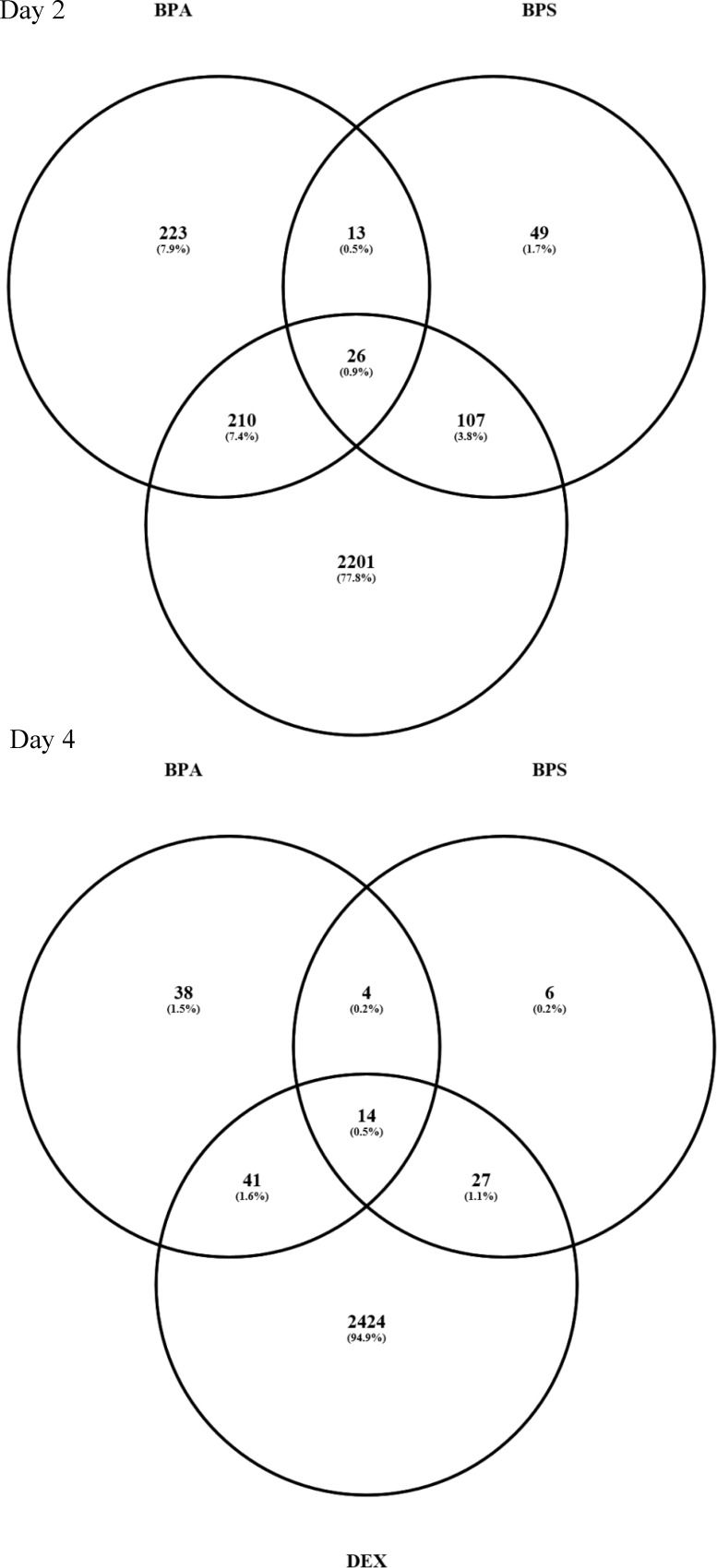
BPA, BPS and DEX treatments result in common and unique DEGs. The Venn diagram is showing overlap of significantly differentially expressed genes (-1.5 ≥fold change ≥ 1.5, FDR p ≤ 0.05) in human preadipocytes treated with BPA, BPS or DEX on Day 2 and Day 4. Venn diagrams were produced using the on-line tool Venny (http://bioinfogp.cnb.csic.es/tools/venny/).

Changes in the transcriptional response of differentiating preadipocytes treated with BPA, BPS or DEX relative to vehicle control, in the presence of IBMX, insulin and troglitazone, were identified by poly-A RNA-seq. DEX-treated cells were used as a positive control, as DEX is a known potent activator of adipogenesis in human primary preadipocytes [28]. The number of DEGs on day 2 and day 4 is summarized in **Fig 1**. For BPA-treated cells, changes in gene expression were observed for 472 genes on day 2 and 176 genes on day 4. For BPS-treated cells, changes in gene expression were observed for 195 genes on day 2 compared to only 51 genes on day 4. Significant changes in gene expression were observed for 2544 genes in response to DEX on day 2 and 2469 genes on day 4. Of the DEGs affected by BPS on day 2, only 39 genes (20%) were in common with BPA (**Fig 1**), whereas 68% of the DEGs changed by BPS were in common with DEX. For BPA treatment 50% of the DEGs were also altered by exposure to DEX. Likewise on day 4, 19 DEGs (40%) following BPS-treatment were also altered by BPA, whereas 80% of the BPS-induced DEGs were in common with DEX. In contrast, only 55% of the DEGs following BPA treatment were in common with DEX on day 4. Interestingly and perhaps unexpectedly, the data show that BPS induces more DEGs in common with DEX than it does with BPA.

When the top ten DEGs following treatment with BPA or BPS were compared surprisingly, none of the top regulated genes were common between the two compounds (Table 1). On day 2, the largest fold-increase in gene expression following BPA treatment was ecotropic viral integration site 2B (*EVI2B*) (5.7-fold), while the most down-regulated gene was grainyhead-like 1 (*GRHL1*) (-3.5-fold). The largest fold-increase in gene expression following BPS treatment was fatty acid binding protein (*FABP)* 4 *4* (6.9-fold). Other notable up-regulated DEGs by BPS treatment on day 2 included *FABP5* (4.8-fold), Glycerol-3-Phosphate Dehydrogenase 1 *(GPD1)* (3.6-fold), Perilipin-4 *(PLIN4)* (3.4-fold) and cell death inducing DFFA like effector C *(CIDEC)* (2.9-fold), all genes involved in lipid metabolism. The most down-regulated gene following treatment with BPS on day 2 was autophagy related 9B (*ATG9B*) (-4.0-fold). In cells treated with DEX, the largest fold-increase in gene expression was phosphoenolpyruvate carboxykinase 1 *(PCK1)* (199.4-fold). Other notable gene changes in the DEX treatment group included adiponectin *(ADIPOQ)* (107.0-fold), *CIDEC* (107.0-fold), *GPD1* (60.9-fold) and perilipin 1 (*PLIN1*) (42.6-fold), consistent with the fact that DEX is a well-known regulator of lipid metabolism and adipogenesis [18, 28, 29]. The complete list of DEGs in response to BPA, BPS and DEX is shown in **S1–S3 Tables**. The data suggest that BPS modulates DEGs specifically related to lipid metabolism and adipogenesis to a greater extent than BPA.

**Table 1:** Top differentially expressed genes with the largest fold changes on day 2 and day 4 following treatment with BPA or BPS. False Discovery Rate (FDR)<0.05, -1.5 ≤ Fold-Change >1.5.

**a) BPA Day 2**				
**Gene symbol**	**Gene name**	**GO biological process**	**Fold change**	**FDR**
*EVI2B*	Ecotropic Viral Integration Site 2B	Integral component of plasma membrane	5.7	3.10x10^-8^
*IL1RN*	Interleukin 1 Receptor Antagonist	Interleukin-1 receptor binding	5.6	1.49x10^-7^
*OMG*	Oligodendrocyte-myelin glycoprotein	Protein binding	4.6	3.24x10^-34^
*MCF2L*	MCF.2 Cell Line Derived Transforming Sequence-Like	Rho guanyl-nucleotide exchange factor activity	4.2	8.20x10^-17^
*PKD1L2*	Polycystic Kidney Disease 1-Like 2	Ion channel activity	3.8	1.63x10^-5^
*IL4I1*	Interleukin 4 Induced 1	L-amino-acid oxidase activity	3.5	3.84x10^-4^
*TIGD7*	Tigger Transposable Element Derived 7	Nucleic acid binding	3.5	8.72x10^-4^
*STON2*	Stonin 2	Intracellular protein transport	3.4	4.68x10^-4^
*MTSS1*	Metastasis Suppressor 1	Movement of cell or subcellular component	3.3	7.88x10^-14^
*RP11-184M15*.*1*		Known lincRNA	-4.4	7.52x10^-22^
*GRHL1*	Grainyhead-Like 1	RNA polymerase II core promoter proximal region sequence-specific DNA binding	-3.5	2.08x10^-3^
*LIFR-AS1*	LIFR Antisense RNA 1	Unknown	-3.5	1.58x10^-3^
*OLAH*	Oleoyl-ACP Hydrolase	Oleoyl-[acyl-carrier-protein] hydrolase activity	-3.5	2.39x10^-4^
*RP11-61L23*.*2*		Known transcribed unprocessed pseudogene	-3.4	2.84x10^-3^
*AMZ1*	Archaelysin Family Metallopeptidase 1	Metallopeptidase activity	-3.2	2.29x10^-4^
*MAMDC2*	MAM Domain Containing 2	Glycosaminoglycan binding	-3.2	2.19x10^-9^
*SDPR*	Serum Deprivation Response	Phosphatidylserine binding	-3.1	1.28x10^-15^
*HIST1H4K*	Histone Cluster 1, H4k	Chromatin organization	-2.8	1.77x10^-4^
*AC009404*.*2*		Known lincRNA	-2.8	3.32x10^-5^
**b) BPS Day 2**				
**Gene symbol**	**Gene name**	**GO biological process**	**Fold change**	**FDR**
*FABP4*	Fatty Acid Binding Protein 4	Transporter activity	6.9	5.13x10^-12^
*FABP5*	Fatty Acid Binding Protein 5	Transporter activity	4.8	1.57x10^-12^
*GPD1*	Glycerol-3-Phosphate Dehydrogenase 1	Glycerol-3-phosphate dehydrogenase activity	3.6	1.57x10^-7^
*SCDP1*	Stearoyl-CoA Pseudogene 1	Unknown	3.5	4.78x10^-3^
*PLIN4*	Perilipin 4	Lipid particle	3.4	4.21x10^-27^
*TSPAN15*	Tetraspanin 15	Plasma membrane, protein binding	3.1	4.76x10^-8^
*CIDEC*	Cell Death-Inducing DFFA-Like Effector C	Lipid particle formation	2.9	1.46x10^-7^
*LGALS12*	Lectin, Galactoside-Binding, Soluble, 12	Regulation of fat cell differentiation	2.9	1.08x10^-3^
*FABP3*	Fatty Acid Binding Protein 3	Transporter activity	2.8	9.82x10^-6^
*FAM151B*			2.6	4.42x10^-2^
*ATG9B*	Autophagy Related 9B	Autophagic vacuole membrane	-4.0	4.47x10^-6^
*KLF3-AS1*	KLF3 Antisense RNA 1	Unknown	-3.2	4.57x10^-4^
*TFR2*	Transferrin Receptor 2	Transferrin receptor activity	-2.9	5.71x10^-5^
*DHRS13*	Dehydrogenase/Reductase (SDR Family) Member 13	Oxidoreductase activity	-2.6	1.90x10^-4^
*TECTA*	Tectorin Alpha	protein binding	-2.6	1.21x10^-3^
*SLC9B1*	Solute Carrier Family 9, Subfamily B Member 1	Solute:proton antiporter activity	-2.5	4.52x10^-3^
*MANEAL*	Mannosidase, Endo-Alpha-Like	Hydrolase activity	-2.5	1.42x10^-2^
*PDE2A*	Phosphodiesterase 2A	cAMP catabolic process	-2.5	2.55x10^-2^
*CCL28*	Chemokine (C-C Motif) Ligand 28	Chemokine activity	-2.4	6.87x10^-4^
*CTC-429P9*.*5*		Known sense intronic	-2.3	1.00x10^-2^
**c) BPA Day 4**				
**Gene symbol**	**Gene name**	**GO biological process**	**Fold change**	**FDR**
*SLC2A4*	Solute Carrier Family, Member 4	Glucose transmembrane transporter activity	4.6	3.69x10^-5^
*MKLN1-AS*	Muskelin 1 Antisense RNA	Unknown	3.8	1.24x10^-3^
*CNIH3*	Cornichon Protein 3	Intracellular signal transduction	2.9	1.89x10^-4^
*SUGT1P3*	SUGT1 Pseudogene 3	Unknown	2.9	3.29x10^-2^
*FERMT1*	Fermitin Family Member 1	Cell adhesion	2.8	1.02x10^-2^
*ARG2*	Arginase 2	Arginine metabolic process	2.8	1.46x10^-3^
*NDUFA6-AS1*	NADH Dehydrogenase (Ubiquinone) 1 Alpha Subcomplex, 6 Antisense RNA 1	Unknown	2.7	1.39x10^-3^
*SCDP1*	Stearoyl-CoA Desaturase Pseudogene 1	Unknown	2.6	3.32x10^-3^
*ALPK3*	Alpha-Kinase 3	Protein serine/threonine kinase activity	2.6	1.12x10^-2^
*YWHAZP2*	Tyrosine 3-Monooxygenase/Tryptophan 5-Monooxygenase Activation Protein, Zeta Pseudogene 2	Unknown	2.6	5.04x10^-2^
*AP000580*.*1*		Known lincRNA	-4.0	5.91x10^-4^
*SPATA41*	Spermatogenesis Associated 41 (non-protein coding)		-3.7	1.24x10^-3^
*CXCL6*	Chemokine (C-X-C Motif) Ligand 6	Chemotaxis	-3.3	7.81x10^-8^
*CCL11*	Chemokine (C-C Motif) Ligand 11	Chemokine activity	-3.0	2.92x10^-12^
*RP11-834C11*.*7*		Known lincRNA	-3.0	6.56x10^-5^
*MT1F*	Metallothionein 1F	Cellular response to cadmium ion	-2.9	1.33x10^-4^
*CA8*	Carbonic Anhydrase VIII	Carbonate dehydratase activity	-2.7	2.76x10^-2^
*LIPM*	Lipase, Family Member M	Lipid metabolic process	-2.5	3.36x10^-3^
*GVQW1*	GVQW Motif Containing 1	Unknown	-2.5	2.14x10^-2^
*HEYL*	Hes-Related Family BHLH Transcription Factor With YRPW Motif-Like	Protein binding transcription factor activity	-2.5	1.06x10^-2^
**d) BPS Day 4**				
**Gene symbol**	**Gene name**	**GO biological process**	**Fold change**	**FDR**
*THRSP*	Thyroid Hormone Responsive	Regulation of lipid biosynthetic process	3.8	9.59x10^-5^
*ALDH1L1*	Aldehyde Dehydrogenase 1 Family, Member L1	Aldehyde dehydrogenase (NAD) activity	3.7	1.78x10^-3^
*ACSM5*	Acyl-CoA Synthetase Medium-Chain Family Member 5	Fatty acid metabolic process	3.4	1.16x10^-3^
*FHDC1*	FH2 Domain Containing 1	Unknown	3.0	2.85x10^-2^
*TMEM132C*	Transmembrane Protein 132C	Integral component of membrane	2.7	4.57x10^-3^
*RP11-44N21*.*1*		Known lincRNA	2.6	3.38x10^-2^
*SEMA3G*	Semaphorin 3G	Multicellular organismal development	2.5	4.44x10^-3^
*PCK1*	Phosphoenolpyruvate Carboxykinase 1	Carbohydrate metabolic process	2.4	2.92x10^-3^
*DLGAP5*	Discs, Large Homolog-Associated Protein 5	Mitotic chromosome movement towards spindle pole	2.4	1.59x10^-2^
*TMEM176B*	Transmembrane Protein 176B	Cell differentiation	2.3	4.52x10^-3^
*ANXA3*	Annexin A3	Calcium-dependent phospholipid binding	-3.7	7.29x10^-3^
*TPI1P2*	Triosephosphate Isomerase 1 Pseudogene 2	Unknown	-2.7	2.50x10^-2^
*FAM115C*	Family With Sequence Similarity 115, Member C	Ion channel binding	-2.6	1.18x10^-3^
*RP11-573G6*.*4*		Unknown	-2.6	4.42x10^-2^
*MESP2*	Mesoderm Posterior Basic Helix-Loop-Helix Transcription Factor 2	Signal transduction involved in regulation of gene expression	-2.5	3.73x10^-2^
*UBL7-AS1*	Ubiquitin-Like 7 Antisense RNA 1	Unknown	-1.9	4.42x10^-2^
*TLL1*	Tolloid-Like 1	Extracellular matrix organization	-1.8	4.01x10^-3^

### Validation of select differentially expressed genes by real-time quantitative PCR (RT-qPCR)

**Fig 2:**
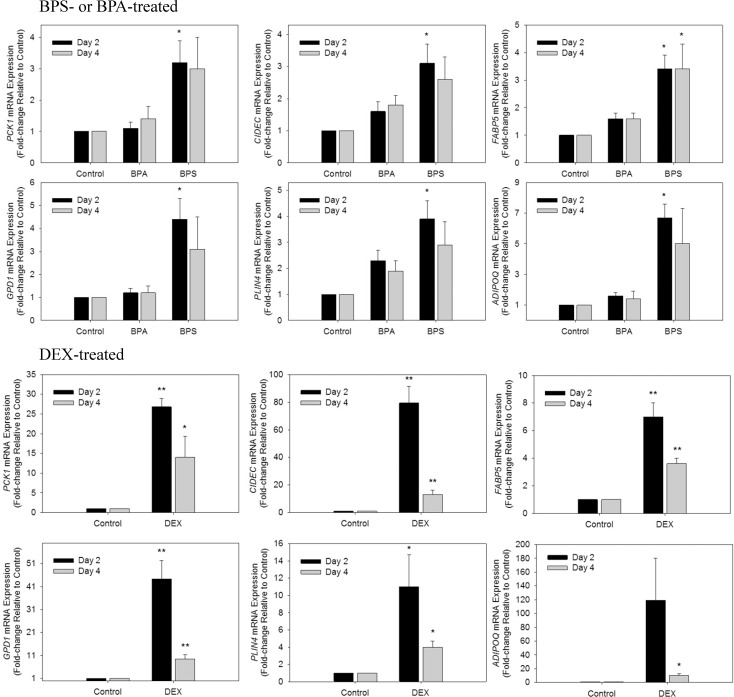
RT-qPCR confirmation of mRNA expression of select genes identified in the RNA-seq analysis. Human primary preadipocytes were treated with BPA, BPS or DEX as described in Material and Methods. Total RNA was isolated 2 and 4 days post-treatment and used for RT-qPCR analysis of the indicated genes normalized to ACTB. Values are expressed as mean fold change relative to control +/- SEM for five experiments. *p < 0.05, **p < 0.001 (n = 5) relative to control calculated by ANOVA.

Expression changes for six genes affected in the RNA-seq following exposure to BPA, BPS or DEX and known to be involved in adipogenesis, were validated by RT-qPCR (**Fig 2**). The fold-changes in gene expression following treatment were mostly consistent with the RNA-seq analysis. Some of the gene changes in BPS-treated cells identified by RT-qPCR were not identified as significantly changed by RNA-seq, namely *PCK1* and *ADIPOQ* on day 2 as well as *FABP5* and *PLIN4* on day 4. This is likely due to the differences in sensitivity between the two techniques. Remarkably, for all the genes validated which include *PCK1*, *CIDEC*, *FABP5*, *GPD1*, *PLIN4* and *ADIPOQ*, BPS was much more efficient than BPA at upregulating these genes. Further the extent of the induction was greater and reached statistical significance for BPS treatment, with fold changes in expression levels as compared to controls reaching 3, 3, 4, 5, 4 and 7-fold respectively (**Fig 2**). Interestingly, the fold increases, at day 4, in response to BPS was comparable to the fold increases in response to DEX, our potent positive control (**Fig 1**).

### Canonical pathways identified in IPA’s Knowledge Base

The DEGs after each of the treatments i.e. BPA, BPS and DEX were uploaded into IPA, and canonical pathways (curated pathways with or without specific directionality) that were over-represented (enriched) within the dataset are presented in Table 2. All pathways presented were statistically significantly altered in IPA. Pathways that were the most significantly enriched with DEGs from BPA and BPS treatment groups are listed in **Table 2**. The most perturbed pathways in BPA-treated preadipocytes on day 2 included hepatic fibrosis, liver X receptor/retinoid X receptor (LXR/RXR) activation and hepatic cholestasis. Pathways found to be perturbed by BPA treatment on day 4 included LXR/RXR activation and atherosclerosis signaling. The top pathways, by statistical significance, in BPS-treated preadipocytes on day 2 include 5' adenosine monophosphate-activated protein kinase (AMPK) signaling, adipogenesis and cholesterol biosynthesis. DEG enriched BPS-associated pathways on day 4 included LXR/RXR activation, AMPK signaling and peroxisome proliferator-activated receptor alpha (PPARA)/RXRA activation, pathways that are related to lipid metabolism and/or adipogenesis. The complete list of pathways identified following BPA, BPS and DEX treatment is shown in **S4–S6 Tables**. Much like the differences in DEGs, BPS-treatment affected a greater number of pathways involved in lipid metabolism and adipogenesis than BPA-treatment.

**Table 2:** Top Canonical pathways identified by IPA on day 2 and day 4 following treatment with BPA or BPS (p<0.05).

**a) BPA Day 2**		
**IPA Canonical Pathway**	**p-value**^**1**^	**Ratio**^**2**^
Hepatic Fibrosis/Hepatic Stellate Cell Activation	8.91E-08	0.090
LXR/RXR Activation	1.41E-04	0.0811
Hepatic Cholestasis	3.98E-04	0.0645
Granulocyte Adhesion and Diapedesis	1.55E-03	0.0584
Dopamine-DARPP32 Feedback in cAMP Signaling	1.78E-03	0.0573
Role of Osteoblasts, Osteoclasts and Chondrocytes in Rheumatoid Arthritis	4.79E-03	0.0463
Serotonin Receptor Signaling	5.01E-03	0.1
Gap Junction Signaling	5.25E-03	0.0526
Agranulcyte Adhesion and Diapedesis	8.32E-03	0.0488
Prostanoid Biosynthesis	1.00E-02	0.222
Renin-Angiotensin Signaling	1.17E-02	0.0556
Clathrin-mediated Endocytosis Signaling	1.20E-02	0.0457
Atherosclerosis Signaling	1.55E-02	0.0522
**b) BPS Day 2**		
**IPA Canonical Pathway**	**p-value**^**1**^	**Ratio**^**2**^
AMPK Signaling	1.82E-04	0.04
Hepatic Fibrosis / Hepatic Stellate Cell Activation	1.95E-04	0.0395
Adipogenesis Pathway	2.57E-04	0.0458
Superpathway of Cholesterol Biosynthesis	8.71E-04	0.107
Role of NANOG in Mammalian Embryonic Stem Cell Pluripotency	8.91E-04	0.0455
Mitotic Roles of Polo-like Kinase	9.12E-04	0.0625
Basal Cell Carcinoma Signaling	1.26E-03	0.0571
Cholesterol Biosynthesis I	3.31E-03	0.154
Cholesterol Biosynthesis II	3.31E-03	0.154
Cholesterol Biosynthesis III	3.31E-03	0.154
Nitric Oxide Signaling in the Cardiovascular System	4.17E-03	0.0412
HIF1A Signaling	4.79E-03	0.0396
Axonal Guidance Signaling	8.13E-03	0.0188
Role of Pattern Recognition Receptors in Recognition of Bacteria and Viruses	8.32E-03	0.0339
RAR Activation	8.91E-03	0.0266
**c) BPA Day 4**		
**IPA Canonical Pathway**	**p-value**^**1**^	**Ratio**^**2**^
Agranulocyte Adhesion and Diapedesis	8.13E-04	0.0366
LXR/RXR Activation	8.91E-04	0.045
Role of IL-17F in Allergic Inflammatory Airway Diseases	2.40E-03	0.075
Oleate Biosynthesis II	2.82E-03	0.167
Hepatic Fibrosis/Hepatic Stellate Cell Activation	6.76E-03	0.0282
Atherosclerosis Signaling	7.41E-03	0.0348
Growth Hormone Signaling	1.02E-02	0.0448
**BPS Day 4**		
**IPA Canonical Pathway**	**p-value**^**1**^	**Ratio**^**2**^
LXR/RXR Activation	1.35E-04	0.036
AMPK Signaling	7.76E-04	0.0229
PPARA/RXRA Activation	7.94E-04	0.0227
Renin-Angiotensin Signaling	2.14E-03	0.0278
FXR/RXR Activation	2.69E-03	0.0256
Neuroprotective Role of THOP1 in Alzheimer’s Disease	3.09E-03	0.0571
Biotin-carboxyl Carrier Protein Assembly	7.08E-03	0.33
Glycerol-3-phosphate Shuttle	7.08E-03	0.33
PXR/RXR Activation	1.05E-02	0.0308
GPCR-Mediated Integration of Enteroendocrine Signaling	1.12E-02	0.0294
Leptin Signaling in Obesity	1.29E-02	0.0274

^1^P-Values calculated using Fisher-extract test by IPA.

^2^Ratio represents the number of differentially expressed genes mapped to the IPA pathway divided by the total number of genes in the IPA pathway.

### Disease and biological function enrichment analysis

IPA was also used to investigate the potential links to biological outcomes and disease, based on DEGs enrichments and containing high quality gene ontology information and curated lists of genes from the literature linked to specific biological functions and diseases. The summary of the analysis is shown in **Table 3**. The top biological functions affected in BPA-treated differentiating preadipocytes on day 2 were associated with embryonic development and pressure of arteries, while the top two biological functions enriched in BPA-treated preadipocytes on day 4 were associated with lipid metabolism (concentration of triacylglycerol), and cell death and survival related to muscle. The biological functions enriched in BPS-treated preadipocytes on day 2 were primarily related to lipid metabolism including uptake and storage of lipid, quantity of adipose tissue and transport of lipid and fatty acids as well as weight gain. Similarly, the disease and biological functions enriched in BPS-treated preadipocytes on day 4 were also associated with lipid metabolism, fat accumulation and obesity. The complete list of diseases and functions enriched following treatment with BPA and BPS on day 4 was well as DEX on both day 2 and day 4 are shown in **S7–S9 Tables**, respectively.

**Table 3:** Top Biological Functions and Diseases identified through IPA on day 2 and day 4 following treatment with BPA or BPS (p<0.05).

**a) BPA Day 2**				
**Disease or Functions Annotation**	**P-value**		**Predicted Activation State**	**Activation z-score**
Embryonic development, development of body trunk	4.22x10^-5^		Increased	2.634
Pressure of artery	3.23x10^-5^		Increased	2.204
Quantity of nitric oxide	2.49x10^-4^		Increased	2.131
Thrombosis	3.21x10^-8^		Decreased	-2.588
Cell movement of dermal cells	3.44x10^-6^		Decreased	-2.178
Organismal death	2.12x10^-4^		Decreased	-2.055
Infection of mammalia	8.51x10^-4^		Decreased	-2.036
**b) BPS Day 2**				
**Disease or Functions Annotation**	**P-value**		**Predicted Activation State**	**Activation z-score**
Transport of lipid	1.05x10^-4^		Increased	2.582
Cellular infiltration by granulocytes	3.29x10^-3^		Increased	2.433
Epithelial cancer	2.45x10^-5^		Increased	2.430
Cancer	1.62x10^-4^		Increased	2.405
Digestive organ tumor	1.93x10^-3^		Increased	2.362
Malignant solid tumor	4.67x10^-4^		Increased	2.314
Neoplasia of epithelial tissue	6.59x10^-7^		Increased	2.282
Infiltration of neutrophils	4.21x10^-3^		Increased	2.236
Storage of lipid	3.39x10^-5^		Increased	2.200
Transport of long chain fatty acid	3.89x10^-7^		Increased	2.196
Uptake of lipid	6.13x10^-6^		Increased	2.196
Transport of carboxylic acid	7.60x10^-6^		Increased	2.195
Digestive system cancer	3.14x10^-3^		Increased	2.195
Synthesis of steroid	4.65x10^-4^		Increased	2.194
Quantity of adipose tissue	2.18x10^-4^		Increased	2.140
Weight gain	3.33x10^-4^		Increased	2.139
Synthesis of lipid	1.82x10^-6^		Increased	2.132
Insulin sensitivity	4.31x10^-4^		Decreased	-2.412
Ploidy	9.63x10^-4^		Decreased	-2.216
**c) BPA Day 4**				
**Disease or Functions Annotation**	**P-value**		**Predicted Activation State**	**Activation z-score**
Necrosis of muscle	3.51x10^-4^		Increased	2.942
Cell death of muscle cells	1.26x10^-3^		Increased	2.804
Quantity of adipose tissue	4.34x10^-3^		Increased	2.426
Cell death of heart	2.26x10^-3^		Increased	2.401
Quantity of lymph follicle	2.98x10^-3^		Increased	2.156
Concentration of triacylglycerol	5.74x10^-7^		Increased	2.011
Ion homeostasis	3.85x10^-3^		Decreased	-2.425
Angiogenesis	1.01x10^-3^		Decreased	-2.415
Insulin sensitivity	2.81x10^-3^		Decreased	-2.219
Size of bone	3.67x10^-4^		Decreased	-2.217
Ingestion by rodents	9.01x10^-4^		Decreased	-2.213
**d) BPS Day 4**				
**Disease or Functions Annotation**	**P-value**		**Predicted Activation State**	**Activation z-score**
Quantity of adipose tissue	1.55x10^-13^		Increased	2.975
Mass of genitourinary system	1.78x10^-6^		Increased	2.658
Size of body	2.80x10^-4^		Increased	2.594
Fatty acid metabolism	1.34x10^-6^		Increased	2.428
Quantity of white adipose tissue	3.70x10^-9^		Increased	2.425
Mass of testis	1.69x10^-5^		Increased	2.414
Mass of fat pad	3.53x10^-7^		Increased	2.384
Obesity	1.35x10^-8^		Increased	2.372
Mass of epididymal fat	1.71x10^-7^		Increased	2.236
Concentration of triacylglycerol	8.33x10^-13^		Increased	2.152
Concentration of lipid	1.17x10^-11^		Increased	2.125
Consumption of oxygen	6.50x10^-7^		Decreased	-2.425
Energy expenditure	3.98x10^-8^		Decreased	-2.414
Hypoglycemia	1.27x10^-4^		Decreased	-2.000

### Upstream regulator analysis

IPA was also used to predict upstream modulators (including transcription factors, cytokines and chemical exposures) that are consistent with the altered patterns of transcription observed and that might be driving the expression changes. Statistically significant enrichment of upstream regulators of the DEGs was identified for BPA- and BPS-treated cells using IPA (**Table 4**). For BPA treatment, positive upstream regulators of day 2 DEGs included leptin receptor (LEPR), uncoupling protein 1 (UCP1) and follicle stimulating hormone beta (FSHB), whereas negative upstream regulators included IKAROS family zinc finger 1 (IKZF1), serum response factor (SRF) and megakaryoblastic leukemia 1 (MKL1). Positive upstream regulators of DEGs on day 4 for BPA included prostaglandin E receptor 4 (PTGER4) and PPARG, whereas negative upstream regulators included interleukin-1 beta (IL1B), insulin receptor (INSR) and nuclear receptor co-repressor 1 (N-CoR). For BPS treatment, positive upstream regulators for day 2 DEGs included PPARG, PPARA, CCAAT-enhancer-binding protein beta (CEBPB), colony stimulating factor 2 (CSF2), SREBP cleavage-activating protein (SCAP), mediator complex subunit 1 (MED1) and mesenteric estrogen-dependent adipogenesis (MEDAG) whereas the negative upstream regulators included insulin-induced gene 1 (INSIG1) and N-CoR. Interestingly, for BPS, the only positive upstream regulator identified on day 4 was PPARG, whereas negative upstream regulators included N-CoR, homeobox A10 (HOXA10) and mediator complex subunit 13 (MED13). The complete list of the many upstream regulators identified in response to DEX is listed in **S10 Table**.

**Table 4:** Top Upstream Regulators identified through IPA on day 2 and day 4 following treatment with BPA or BPS (p<0.05).

**a) BPA Day 2**				
**Upstream Regulator**	**Molecule Type**	**Predicted Activation State**	**Activation z-score**	**P-value**
LEPR	Transmembrane receptor	Activated	2.197	1.91x10^-4^
UCP1	Transporter	Activated	2.200	6.14x10^-2^
FSHB	Other	Activated	2.000	3.51x10^-4^
ALOX5	Enzyme	Activated	2.000	4.23x10^-4^
IKZF1	Kinase	Inhibited	-2.359	1.80x10^-5^
SRF	Group	Inhibited	-2.190	2.81x10^-2^
MKL1	Enzyme	Inhibited	-2.166	5.19x10^-3^
**b) BPS Day 2**				
**Upstream Regulator**	**Molecule Type**	**Predicted Activation State**	**Activation z-score**	**P-value**
PPARA	Ligand-dependent nuclear receptor	Activated	3.244	4.64x10^-12^
PPARG	Ligand-dependent nuclear receptor	Activated	2.831	2.26x10^-14^
CSF2	Cytokine	Activated	2.818	6.56x10^-6^
MED1	Transcription regulator	Activated	2.219	1.31x10^-5^
MEDAG	Other	Activated	2.000	1.08x10^-7^
SCAP	Other	Activated	2.000	2.89x10^-4^
INSIG1	Other	Inhibited	-2.449	2.89x10^-5^
N-CoR	Group	Inhibited	-2.219	5.15x10^-6^
**c) BPA Day 4**				
**Upstream Regulator**	**Molecule Type**	**Predicted Activation State**	**Activation z-score**	**P-value**
PTGER4	G-protein coupled receptor	Activated	2.449	1.45x10^-4^
RBPJ	Transcription regulator	Activated	2.219	2.62x10^-4^
PPARG	Ligand-dependent nuclear receptor	Activated	2.028	3.80x10^-5^
IL1B	Cytokine	Inhibited	-2.368	1.23x10^-3^
INSR	Kinase	Inhibited	-2.219	1.14x10^-4^
IKBKG	Kinase	Inhibited	-2.219	6.28x10^-4^
TICAM1	Other	Inhibited	-2.216	8.30x10^-4^
N-CoR	Group	Inhibited	-2.213	7.06x10^-5^
Map3k7	Kinase	Inhibited	-2.000	4.69x10^-4^
MAPK9	Kinase	Inhibited	-2.000	2.33x10^-3^
**BPS Day 4**				
**Upstream Regulator**	**Molecule Type**	**Predicted Activation State**	**Activation z-score**	**P-value**
PPARG	Ligand-dependent nuclear receptor	Activated	2.787	1.45x10^-9^
N-CoR	Group	Inhibited	-2.630	1.29x10^-10^
HOXA10	Transcription regulator	Inhibited	-2.000	5.20x10^-5^
MED13	Transcription regulator	Inhibited	-2.000	1.34x10^-7^

Activation z-score +/- 2 indicates a statistically significant directionality in the pathway.

### Regulator network analysis

**Fig 3:**
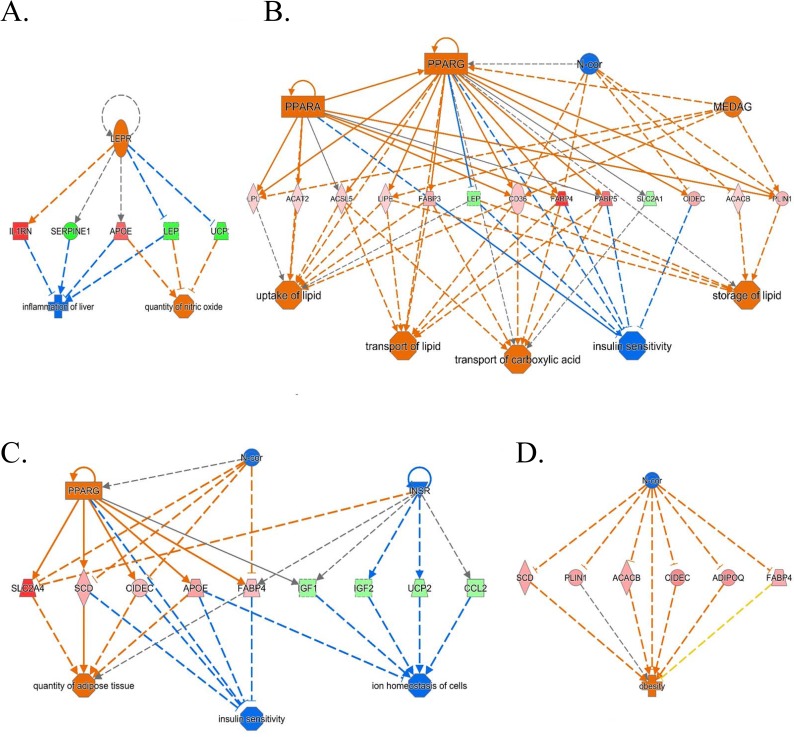
Regulatory network analysis using IPA. Regulatory network generated in IPA for genes that were differentially expressed following exposure to BPA (A, B) and BPS (C, D) on day 2 and day 4. Orange represents activated upstream regulator or biological process; Blue represents inhibited upstream regulator or biological process; Red indicates gene expression is up-regulated; Green indicates gene expression is down-regulated.

Networks that were assigned a p-score (defined as–log(p-value)) based on a Fisher’s exact test using the number of genes in the network and the number of focus genes in the network relative to IPA’s ‘Global Molecular Network’ are also presented in Fig 3. This analysis revealed a main regulatory network for BPA on day 2 with LEPR as an upstream regulator for a number of genes linked to inflammation of the liver and quantity of nitric oxide (**Fig 3A**). Interestingly, in BPS-treated cells on day 2 the regulatory network shown in **Fig 3B** identified key adipogenic factors (PPARA, PPARG, MEDAG and N-CoR) as upstream regulators that control a group of important lipid metabolism and adipogenesis-related genes including *FABP3*, *FABP4*, cluster of differentiation 36 (*CD36)*, hormone sensitive lipase (*LIPE)*, *FABP5*, acetyl-CoA carboxylase-beta (*ACACB)*, lipoprotein lipase (*LPL)*, *PLIN1* and *CIDEC*. Most of these genes were also part of a network involved in transport of lipid, uptake of lipid, storage of lipid, transport of carboxylic acid and insulin sensitivity. Likewise, BPA treatment altered a network of genes on day 4 related to adipogenesis/lipid metabolism. This network identified the key upstream regulators PPARG and N-CoR and exhibited a small number of genes involved in lipid metabolism which include stearoyl-CoA desaturase (*SCD)*, *CIDEC*, apolipoprotein E (*APOE)* and *FABP4* (**Fig 3C**). On day 4, the network showed inhibition of the upstream regulator N-CoR in BPS-treated cells which leads to activation of adipogenic genes such as *SCD*, *PLIN1*, *ACACB*, *CIDEC*, *ADIPOQ* and *FABP*, leading to obesity (**Fig 3D).**

## Discussion

It is well accepted that exposure to some chemicals, collectively referred to as obesogens, can have repercussions on human health as endocrine disrupters and as possible contributors to the rising obesity rates and metabolic disorders. We and others have previously shown that both BPA and BPS can induce adipogenesis in human and mouse preadipocyte cell models, categorizing them as potential obesogens [17–21]. However, it is not clear whether both compounds act similarly, have the same gene signature, or have the same predicted mode of action in these model systems. Due to the similarities in chemical structure, it was expected that the gene expression profiles after BPA and BPS treatments would be somewhat similar. However, striking differences in the DEGs affected by the two chemicals and particularly those involved in lipid metabolism and adipogenesis were observed in the human pre-adipocytes exposed to the same concentrations of BPA and BPS. Out of the 650 DEGs affected by BPA at day 2 and the 174 DEGs affected by BPA at day 4 only a few DEGs were genes identifiable with the adipocyte phenotype (**S1 Table**). Interestingly, hydroxysteroid 11-beta dehydrogenase 1 (*HSD11B1)*, the enzyme that converts cortisone to cortisol, was up-regulated by BPA treatment consistent with what was previously reported for BPA in adipose tissue from obese children {{204 Wang,J. 2013}}, This indicated that BPA may be acting by increasing local concentrations of active glucocorticoid *in vivo*. Similarly, BPS also increased the expression of *HSD11B1*, in this study, suggesting a possible effect of BPS on the glucocorticoid receptor and on visceral obesity and the adipose tissue *in vivo*. BPS-treatment resulted in many DEGs related to lipid metabolism and adipogenesis, as early as 48 hours post treatment as listed in **Table 1** and **S1 Table**. This suggests that BPS is more specific in its effects and upregulates genes that are specifically related to the development of the adipose tissue and lipid accumulation in the human primary preadipocytes. We have previously reported that in the 3T3-L1 murine model BPS was more potent at inducing adipogenesis than BPA, when compared at equivalent concentrations [23]. In that study we show that BPS was more able than BPA to induce fat accumulation and gene expression of adipogenic markers [23]. In agreement with the mouse model, BPS primarily induced the expression of lipid metabolism related genes and the induction occurred earlier in the differentiation process. In line with this observation, on day 2, there was up-regulation of the fatty acid binding family of proteins showing the conversion of the preadipocytes to mature adipocytes in response to BPS but not BPA treatment. In contrast, adipose specific genes were upregulated only at day four in response to BPA, and there were less adipose specific genes affected. These results show that BPA may be less efficient at promoting adipogenesis than BPS. Further, we also show that BPS induced the expression of all the adipogenic genes confirmed by RT-PCR (**Fig 2**) to double the levels of the ones induced by BPA treatment and interestingly, for some genes, such as ADIPOQ, to the same extent as DEX (**Fig 2**). Again, these results confirm the phenotype predicted by the transcriptomic analysis showing a much more adipose specific phenotype resulting from BPS treatment. Perhaps earlier time points would have shed light on potential transcription factors that may be involved in BPS induced adipogenesis in human primary preadipocytes. One possible mechanism is through PPARG activation as previously shown by our group [23]. PPARG is required for adipogenesis and was identified as an upstream regulator in IPA following BPS-treatment on day 2. PPARG was also identified as an upstream regulator for BPA but only on day 4 (**Fig 3**). We have previously shown that BPS but not BPA is a partial agonist for PPARG [23]. Others have also shown that the BPA analogues tetrabromobisphenol A (TBBPA) and tetrachlorobisphenol A (TCBPA) bind PPARG and promote adipocyte differentiation [30]. PPARG has been found to be an upstream regulator in the RNA-seq analysis corroborating a role for PPARG in the effects mediated. However, we could not detect *PPARG* in the DEGs in either of the chemicals, showing that these PPAR target genes may have been upregulated by another yet unknown regulator, or by activation of PPARG by endogenous ligands. Interestingly, *PPARG* was upregulated by DEX showing that it is unlikely that these chemicals exert their effects through upregulation of PPARG expression, but rather through direct or indirect receptor activation.

One other mechanism of action may be through the recently characterized *ADIRF* which was up-regulated by both BPA and BPS at day 4 and has been shown to play a role in fat cell development by enhancing the expression levels of the regulators of adipocyte differentiation PPARG and CCAAT-enhancer-binding protein alpha (CEBPA) in 3T3 L1 cells [31]. Interestingly, *ADIRF* is also highly expressed in obese individuals, suggesting a role for this gene in the development of obesity [31]. However, it is unlikely that this gene is the initiator of adipogenesis due to its late expression. Future studies will examine the effect of these bisphenols on *ADIRF* expression and its role in regulating chemically induced adipogenesis. Another interesting DEG that was up-regulated in both BPA- and BPS-treated cells was *CIDEC*. Several studies have shown that *CIDEC* is up-regulated during adipogenesis and is localized to the lipid droplet surface where it interacts with PLIN1, which was also affected in this study, and is essential for the differentiation of adipose tissue [32–35]. CIDEC was also shown to bind and cause the degradation of AMPK [36] which was identified as a negative downstream regulator in our analysis in BPS-treated preadipocytes. Inhibition of AMPK was shown to increase SREBF1 activity in hepatocytes [37], and SREBF1 was shown to be a key transcription factor in adipocyte differentiation of 3T3-F442A cells [38]. Regulation of lipid droplet formation and adipogenesis through ADIRF and CIDEC may be common pathways for BPA- and BPS-induced adipogenesis since they were up-regulated by both chemicals in this study.

Despite these few commonalities our analysis showed clear differences between the canonical pathways and upstream regulators identified following BPA- and BPS-treatment. For BPA-treated cells, only a few pathways were related to lipid metabolism and adipogenesis. In contrast, BPS-treated cells revealed alterations in a variety of lipid metabolism/adipogenesis pathways including the adipogenesis pathway, and cholesterol biosynthesis This analysis also suggested that BPS activates a more extensive number of lipogenic and adipogenic pathways and that these changes occur earlier in the differentiation process than following BPA treatment.

There were few interesting findings in the RNA-seq analysis that suggest that BPA and BPS treatments confer unique phenotypes in the resulting adipocyte. For example, BPA but not BPS increased the expression of interleukin 1 receptor antagonist (*IL1RN*) by 5.6 fold. IL1RN has been previously shown to be upregulated in morbidly obese patients and in visceral adipose tissue. IL1RN is a protein that, despite its name, is known to increase inflammation [39] and was also upregulated by DEX by 17 fold (**S3 Table**).

Another discovery in the present study was the perturbation of the LXR/RXR pathway by both BPA (at day 2 and day 4) and BPS at day 4. Despite the fact that this pathway was perturbed, the z-score did not show a clear activation of the pathway, probably due to the limited number of genes involved. It is unlikely that a direct activation of the LXR is involved as Sui Y *et al*. showed that BPA activates PXR but not LXR and therefore the effects of BPA on the LXR/RXR pathway may be indirect [40]. LXR has been shown to increase adipocyte differentiation through PPARG up-regulation [41] [42] and to regulate cholesterol efflux [43]. In this study, LXR was identified as a potential up-stream regulator due to the presence of DEGs such as *APOE* and *SCD* that were previously shown to be affected by LXR activation [44, 45]. However, most of the published data were obtained in hepatocytes, where it has been shown that LXR plays an important role in *de novo* lipogenesis [46]. The role of LXR in adipocytes in general, and especially in human adipocytes is less understood. However, it is known that one of the main downstream target genes of LXR is sterol-regulatory binding factor 1 (SREBF1) which, as previously mentioned, is important in adipogenesis and lipogenesis [47]. Although *SREBF1* expression was not changed, we found that, SCAP was identified as a positive upstream regulator only in BPS-treated preadipocytes at day 2 (**Table 4**). SCAP is crucial for the activation of SREBF1 which in turn is important for lipid accumulation [48]. The PPARA/RXR and FXR and PXR/RXR activation pathways were also identified in BPS-treated preadipocytes and they have all been shown to modulate adipogenesis, lipid metabolism or obesity [42, 49–52]. PXR is not known to be expressed in mouse adipose tissue, either WAT or BAT, [53] however, it is not clear if the same is true for the human primary preadipocytes. Until this is established it is unlikely that PXR has a role in human preadipocyte adipogenesis and unlikely to mediate the effects of BPS.

Interestingly, the N-CoR complex activity was identified to be downregulated in our study both by BPS and BPA (**Fig 3**). At day 4 the only identified pathway linking BPS to obesity was N-CoR inhibition. Knockout of N-CoR in mice adipose tissue resulted in obesity likely through PPARG post-translational regulation [54]. In our study N-Cor inhibition was found by IPA in BPS and BPA treatments and this inhibition could result in obesity, similarly to the findings in N-Cor knockout mice [54].

This study presents the first comparison of the gene expression profiles in BPA- and BPS-treated human preadipocytes early in differentiation. While we showed that both BPA and BPS induce lipid accumulation in these cells [18, 21], the analysis suggests that BPS modulates DEGs and regulatory pathways that are more specific for adipogenesis and lipid metabolism as compared to the broader effects of BPA. Further, through gene expression analysis we show here that BPS treatment, and possibly exposure, resulted in a gene signature that suggests effects on metabolism, adipocity and insulin sensitivity. This study highlights that potential adverse human health effects may result from human exposure to the BPA replacement chemical, BPS, and that these effects warrant further investigation.

## Supporting Information

S1 Table Differentially expressed genes on day 2 and day 4 following treatment with BPA. Data is sorted based on Fold Change (FC). False Discovery Rate (FDR)<0.05, -1.5 < Fold-Change >1.5.
(XLSX)

S2 Table Differentially expressed genes on day 2 and day 4 following treatment with BPS. Data is sorted based on Fold Change (FC). False Discovery Rate (FDR)<0.05, -1.5 < Fold-Change >1.5.
(XLSX)

S3 Table Differentially expressed genes on day 2 and day 4 following treatment with DEX. Data is sorted based on Fold Change (FC). False Discovery Rate (FDR)<0.05, -1.5 < Fold-Change >1.5.
(XLSX)

S4 Table BPA Canonical Pathways. Canonical pathways identified by IPA on day 2 and day 4 following treatment with BPA (p<0.05).
(XLS)

S5 Table BPS Canonical Pathways. Canonical pathways identified by IPA on day 2 and day 4 following treatment with BPS (p<0.05).
(XLS)

S6 Table DEX Canonical Pathways. Canonical pathways identified by IPA on day 2 and day 4 following treatment with DEX (p<0.05).
(XLS)

S7 Table BPA Diseases and Functions. Biological Diseases and function identified by IPA on day 2 and day 4 following treatment with BPA (p<0.05).
(XLS)

S8 Table BPS Diseases and Functions. Biological Diseases and function identified by IPA on day 2 and day 4 following treatment with BPS (p<0.05).
(XLS)

S9 Table DEX Diseases and Functions. Biological Diseases and function identified by IPA on day 2 and day 4 following treatment with DEX (p<0.05).
(XLS)

S10 Table DEX Upstream Regulators. Upstream Regulators identified through IPA on day 2 and day 4 following treatment with DEX (p<0.05).
(XLS)
